# The assembly processes and network characteristics of bacterial, fungal and archaeal communities in the middle Yangtze River and river-connected lakes

**DOI:** 10.3389/fmicb.2025.1701799

**Published:** 2025-10-24

**Authors:** Fenglin Wang, Si Li, Pinjian Li, Chuanzhe Feng, Zhijie Zhao, Yulong Yang, Fulei Han, An Xue, Zhenshan Li, Peng Han

**Affiliations:** ^1^Key Laboratory of Water and Sediment Sciences, Ministry of Education, College of Environmental Sciences and Engineering, Peking University, Beijing, China; ^2^State Environmental Protection Key Laboratory of All Material Fluxes in River Ecosystems, Beijing, China; ^3^Beijing Key Laboratory of Farmland Soil Pollution Prevention and Remediation, College of Resources and Environmental Sciences, China Agricultural University, Beijing, China

**Keywords:** microbial community, environmental DNA, river and river-connected lakes, assembling processes, co-occurrence network

## Abstract

Despite the crucial ecological roles of bacterial, fungal and archaeal communities in rivers and lakes, their interactions and dynamic changes in large, hydrologically-connected river–lake systems remain poorly understood. This study investigated the biogeographic patterns, assembly processes and co-occurrence network characteristics of bacterial, fungal and archaeal communities in the middle reaches of Yangtze River (MYR) and its two largest connected lakes, Dongting Lake (DTL) and Poyang Lake (PYL). Our results revealed significant spatial heterogeneity in microbial diversity and composition, with higher sedimentary microbial diversity in lakes than in the river. Stochastic processes, particularly dispersal limitation, dominated community assembly across all habitats. *β*-NRI analysis showed that deterministic processes were more influential for planktonic bacterial and archaeal communities in the lakes. Co-occurrence network analysis demonstrated that inter-domain cooperation was prevalent in PYL, whereas intra-domain interactions were more common in MYR and DTL, reflecting distinct hydrological connectivity. Keystone taxa differed between rivers and lakes, with rare taxa prevailing in MYR and both rare and abundant taxa contributing in lakes. Our findings highlight how connectivity and flow dynamics fundamentally shape microbial ecology, providing insights into for the management and conservation of large river–lake ecosystems.

## 1 Introduction

Microorganisms play a fundamental role in nutrient transformation, organic matter decomposition and energy flow in river and lake ecosystems (Wu et al., 2013; Staley et al., 2014). The composition of microbial communities in rivers and lakes are influenced by a combination of factors including upstream water inflow, seasonal variations and human activities (Liu et al., 2018). Among different microorganisms, most studies primarily focus on the dynamic changes of bacterial communities in response to environmental pressures (Bai et al., 2020; Liu et al., 2020; Payne et al., 2020; Gao et al., 2021; Zhang Y. et al., 2022; Liu et al., 2024), with little attention paid to other microorganisms such as fungi and archaea. Fungi and archaea are vastly different from bacteria in regard to their morphological traits, growth rates, environmental sensitivities, and substrate utilizations (Hannula et al., 2017). Recently, although the importance of fungal community (Chen et al., 2020; Gu et al., 2024) and archaeal community (Tang et al., 2025) have been reported, the symbiotic patterns of bacterial, fungal, and archaeal communities in changing environments remain poorly understood.

Understanding the intra- and inter-community interactions and assembly mechanisms of bacterial, fungal and archaeal communities is critical for deciphering structure and functional stability (Zhou and Ning, 2017). Most previous studies have focused on microbial communities in river sections, isolated lakes, reservoirs or coastal waters with variations in season and pollutant discharge (Kraemer et al., 2020; Liao et al., 2020; Liu et al., 2022; Zhang T. et al., 2022). In isolated lakes, stronger associations could be formed among bacteria, fungi and archaea under the stronger environmental stress (Zhang et al., 2025). Deterministic processes drive the bacterial (Bai et al., 2020) and archaeal (Tang et al., 2020) community assemblies in isolation lakes. Nevertheless, river and its connected lakes constitute a hydrologically-connected ecosystem with frequent water and sediment exchange (Vannote et al., 1980). Under such dynamic hydrological conditions, the improvement of connectivity significantly intensify the complexity of interactions among bacterial, fungal, and archaeal communities resulting from cooperative and competitive strategies. The differences in the assembly patterns of microbial communities between rivers and river-connected lakes remain poorly understood.

The Yangtze River is the world’s third-longest river and the top longest river in China, spanning 6,363 km with a catchment area exceeding 1.8 million km^2^. The middle reaches of Yangtze River (MYR), joined by its two large lakes [Dongting Lake (DTL) and Poyang Lake (PYL)], constitute one of the most complex and biologically diverse freshwater ecosystems in China (Wang et al., 2019). DTL is a flood basin of the Yangtze River, with strong fluctuation of the water level and surface area at different seasons. PYL, as the largest freshwater lake in China, receives five tributaries from the south and flows northward into the Yangtze River through a channel (Huang et al., 2022). PYL had often experienced reverse flow from the Yangtze River in flood season (Ye et al., 2012). However, the operation of the Three Gorges Dam on the upper Yangtze River in recent years has significant impacts on the water level in MYR (Yang et al., 2019) and its connected lakes (Lai et al., 2014). With the decrease in the riverbed of the Yangtze River, DTL received less water from the Yangtze River while PYL experienced a dramatic recession (Dai and Liu, 2013). These alterations in the hydrological regime will directly affect the interactions of microbial communities in the MYR and its connected lakes, which is of great importance for the balance of the river–lake ecosystem.

In this study, we collected water and sediment samples from MYR, DTL, and PYL, and investigated the dynamic interactions among bacterial, fungal and archaeal communities at high-water and low-water periods. The aims of this study are to (i) elucidate the differences in microbial community diversity and composition between MYR and its connected lakes; (ii) identify the assembly processes and primary driving factors of river–lake microbial communities; (iii) assess the intra- and inter- interactions of bacterial, fungal, and archaeal communities in the river–lake ecosystem.

## 2 Materials and methods

### 2.1 Study area and sample collection

**Figure 1:**
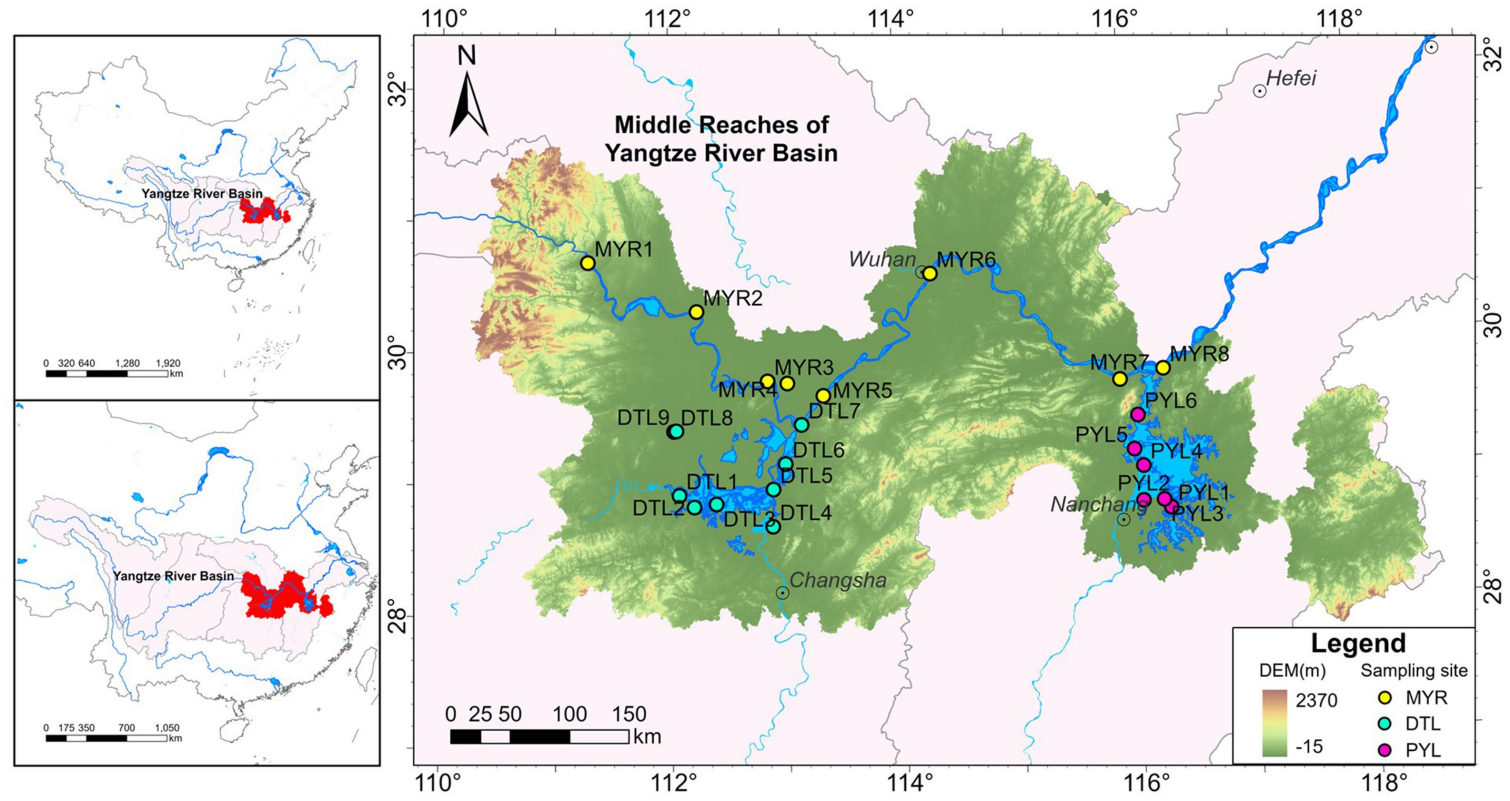
Location of sampling sites in middle reaches of Yangtze River (MYR), Dongting Lake (DTL), and Poyang Lake (PYL).

Water and sediment samples were collected in August 2023 (high-water period) and January 2024 (low-water period) from MYR (MYR1–MYR8), DTL (DTL1–DTL9) and PYL (PYL1–PYL6) (Figure 1; Supplementary Table S1). A total of 45 water samples and 44 sediment samples were obtained. Water and sediment samples from MYR4, and sediment samples from MYR8 were not collected due to the difficulty of collecting in August 2023 (MYR4 is a finless porpoise nature reserve, and we did not obtain an access permit in August 2023. The riverbanks on both sides of the Yangtze River at MYR8 were under construction, and there were no sediments could be collected in August 2023).

At each sampling site, grab sample of 17 L surface water was collected by using brown sterile bottles at 0.5 m below the water surface. Surficial sediment samples were collected by using a Peterson grab in DTL and PYL, and through the lead fish on the hydrographic ship in the MYR with the assistance of the staff from the Changjiang Water Resources Commission. Water and sediment samples were kept on ice and transported to laboratory within 24 h.

Water samples of 12 L were filtered through 0.22 μm hydrophilic nylon membranes (Merck Millipore, USA) for environmental DNA (eDNA) analysis (Li et al., 2018). The 1 L water sample was filtered through a 0.7 μm glass fiber membrane (GF/F, Whatman, UK) for chlorophyll analysis. The membranes were stored at −80 °C until DNA extraction and chlorophyll measurement. Sediment samples for eDNA analysis were instantly put into sterile centrifuge tubes and stored at −80 °C until DNA extraction (Ji et al., 2022). Water sample of 4 L were stored below 4 °C for water quality analyses (Xu et al., 2023).

### 2.2 Measurements of environmental variables

For water samples, 14 environmental variables were measured. All the measurements were completed within 7 days after collecting, following the national standard methods, which were issued by the Ministry of Agriculture and Rural Affairs of the People’s Republic of China and the Ministry of Ecology and Environment of the People’s Republic of China. During field sampling, we measured water temperature, pH, dissolved oxygen (DO), oxidation–reduction potential (Eh) and conductivity on-site with a multi-parameter water quality tester (VZ86031BZ3, AZ Instrument Corp., Taiwan China). Suspended solids (SS, GB 11901-89), permanganate index (COD_Mn_, GB 11892-89), ammonia nitrogen (NH_4_^+^-N, HJ 535-2009), nitrate nitrogen (NO_3_^−^-N, HJ/T 346-2007), nitrite nitrogen (NO_2_^−^-N, HJ/T 346-2007), total nitrogen (TN, HJ 636-2012), total phosphorus (TP, GB 11893-89), total organic carbon (TOC, HJ 501-2009) and chlorophyll-A (Chl, HJ 897-2017) were analyzed in laboratory. For sediment samples, we analyzed in laboratory for pH, NH_4_^+^-N, NO_3_^−^-N, TN, TP and sediment organic carbon (SOC).

### 2.3 PCR amplification, sequencing and bioinformatics analysis

Multiple PCR assays were conducted at a commercial laboratory (Majorbio, Shanghai, China). Briefly, the V3–V4 region of the bacterial 16S rDNA gene was amplified using universal primers 338F (5′-ACTCCTACGGGAGGCAGCA-3′) and 806R (5′-GGACTACHVGGGTWTCTAAT-3′) (Ye et al., 2017), while the primers targeting the V4–V5 region of the archaeal 16 s rDNA gene were 524F10extF (5′-TGYCAGCCGCCGCGGTAA-3′) and Arch958RmodR (5′-YCCGGCGTTGAVTCCAATT-3′) (Chen et al., 2019). The ITS1 region of the fungal ribosomal internal transcribed spacer region was amplified using universal primers ITS1F (5′-CTTGGTCATTTAGAGGAAGTAA-3′) and ITS2R (5′-GCTGCGTTCTTCATCGATGC-3′) (Gill et al., 2020). The PCR program consisted of an initial denaturation at 95 °C for 3 min, followed by 27 cycles of denaturing at 95 °C for 30 s, annealing at 55 °C for 30 s and extension at 72 °C for 45 s, and single extension at 72 °C for 10 min, and end at 4 °C. For each eDNA extract, PCR was performed in triplicate. PCR products were analyzed using 2% agarose gel electrophoresis to confirm successful amplification. Subsequently, the PCR products of the same primers were pooled in equal volumes and purified using the AxyPrep DNA Fragment Purification Kit (Axygen Biosciences, Union City, CA, USA), and quantified using QuantiFluor™-ST (Promega, USA). The samples were normalized to equimolar amounts in the final mixture and sequenced using the strategies of PE250 (paired-end sequenced 250 × 2) on an Illumina MiSeq platform (Majorbio Company in Shanghai).

Raw FASTQ files were de-multiplexed using an in-house perl script, and then quality-filtered by Fastp v0.19.6 (Chen et al., 2018) and merged by FLASH v1.2.11 (Magoc and Salzberg, 2011) with the following criteria (Caporaso et al., 2010). The standards for quality filtering and merging can be found in Supplementary Text S1. The optimized sequences were clustered into operational taxonomic unit (OTU) using UPARSE v11 (Edgar, 2010) with 97% sequence similarity level, and chimeric sequences were identified and removed using UCHIME (Edgar, 2013). The most abundant sequence for each OTU was selected as a representative sequence. The bacterial and archaeal sequences were classified using the SILVA reference database (Release138) (von Hoyningen-Huene et al., 2019), and the taxonomic identity of fungal sequences was queried using the UNITE (Release 8.0) ITS (Chen et al., 2020) reference database. To minimize the effects of sequencing depth on alpha and beta diversity measures, all samples were rarefied based on the lowest sequence depth.

### 2.4 Quantification and statistical analysis

The *α* diversity was calculated using Mothur v1.30.2. The calculation of *β* diversity, nonmetric multidimensional scaling (NMDS) analysis, analysis of similarities (ANOSIM), distance-decay analysis, *β*-net relatedness index (*β*-NRI), Raup-Crick index (RC) were performed using R v4.3.1. The Mann–Whitney U test was conducted using Origin 2024b. The co-occurrence networks were constructed using R v4.3.1, while network rendering and visualization were implemented with Gephi v0.10. Detailed quantification and statistical analysis methods can be found in Supplementary Text S2.

## 3 Results

### 3.1 DNA sequencing results and environmental variables

For all water and sediment samples, a total of 6,621,223 high-quality sequence reads for bacteria, 7,188,460 for fungi and 6,419,729 for archaea were obtained after quality filtering (Supplementary Table S2). These high-quality reads were clustered into 58,522, 25,414, and 14,617 different OTUs for bacteria, fungi and archaea, respectively (Supplementary Figure S1a). 91% OTUs of bacteria, 38% OTUs of fungi and 95% OTUs of archaea were successfully annotated to phylum level. The rarefaction curves showed clear asymptotes (Supplementary Figure S1b), which together indicate a near-complete sampling of the community (Ye et al., 2017). The environmental variables of different sampling sites are shown in Supplementary Figure S2 and Supplementary Table S1.

### 3.2 Alpha and beta diversity

**Figure 2:**
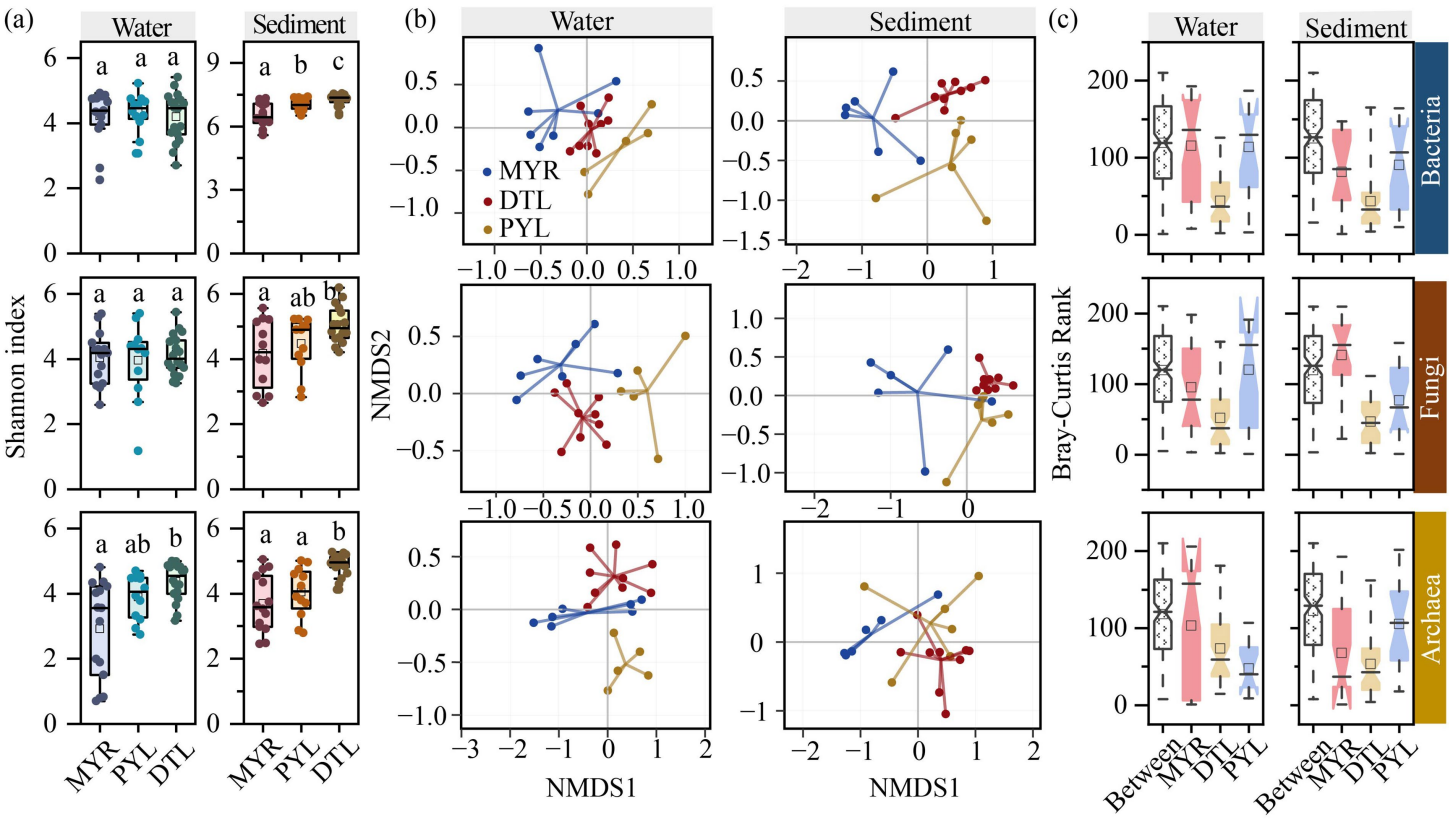
Alpha and beta diversity of bacterial, fungal and archaeal communities in MYR, DTL, and PYL. Shannon index **(a)**. Nonmetric multidimensional scaling (NMDS) analysis based on Bray–Curtis dissimilarity in high-water period **(b)**. ANOSIM analysis in high-water period **(c)**.

Alpha diversities of microbial communities, as evaluated by Ace, Chao1, Coverage, Shannon, Simpson, and Sobs indices, are shown in Supplementary Table S3. Most alpha diversity indices showed consistent statistical trends. Overall, sedimentary microbial diversity in river-connected lakes was significantly higher than in rivers (Supplementary Figure S3). Among different microbial groups, bacteria exhibited significantly higher diversity index than that of fungi and archaea in sediment samples (Supplementary Figure S3), indicating that bacterial communities had the highest diversity and dominated the microbial communities in sediment. Comparing different sampling sites, insignificant difference was observed in planktonic microbial diversity between MYR and its connected lakes, while alpha diversity of bacteria significantly differed in MYR, DTL, and PYL sediment (Figure 2a). In addition, DTL sediment had higher alpha diversity of fungi and archaea than that in MYR and PYL. This result is consistent with the species numbers obtained through high-throughput sequencing across different areas (Supplementary Figure S1a).

NMDS analysis revealed that bacterial, fungal, and archaeal communities had significant compositional variations among MYR, DTL, and PYL in both water and sediment samples (Figure 2b; Supplementary Figure S4a). ANOSIM analysis confirmed the significant differences in bacterial, fungal, and archaeal compositions in MYR and its connected lakes in both high-water period (Figure 2c) and low-water period (Supplementary Figure S4b). In high-water period, the microbial communities in DTL and PYL sediment were more likely to form two separated clusters than that in low-water period. But in low-water period, the planktonic microbial communities from DTL and PYL tended to separate from each other. In MYR, due to the long distance of each sampling sites, the inter-group differences were higher than that in DTL and PYL.

### 3.3 Community composition

**Figure 3:**
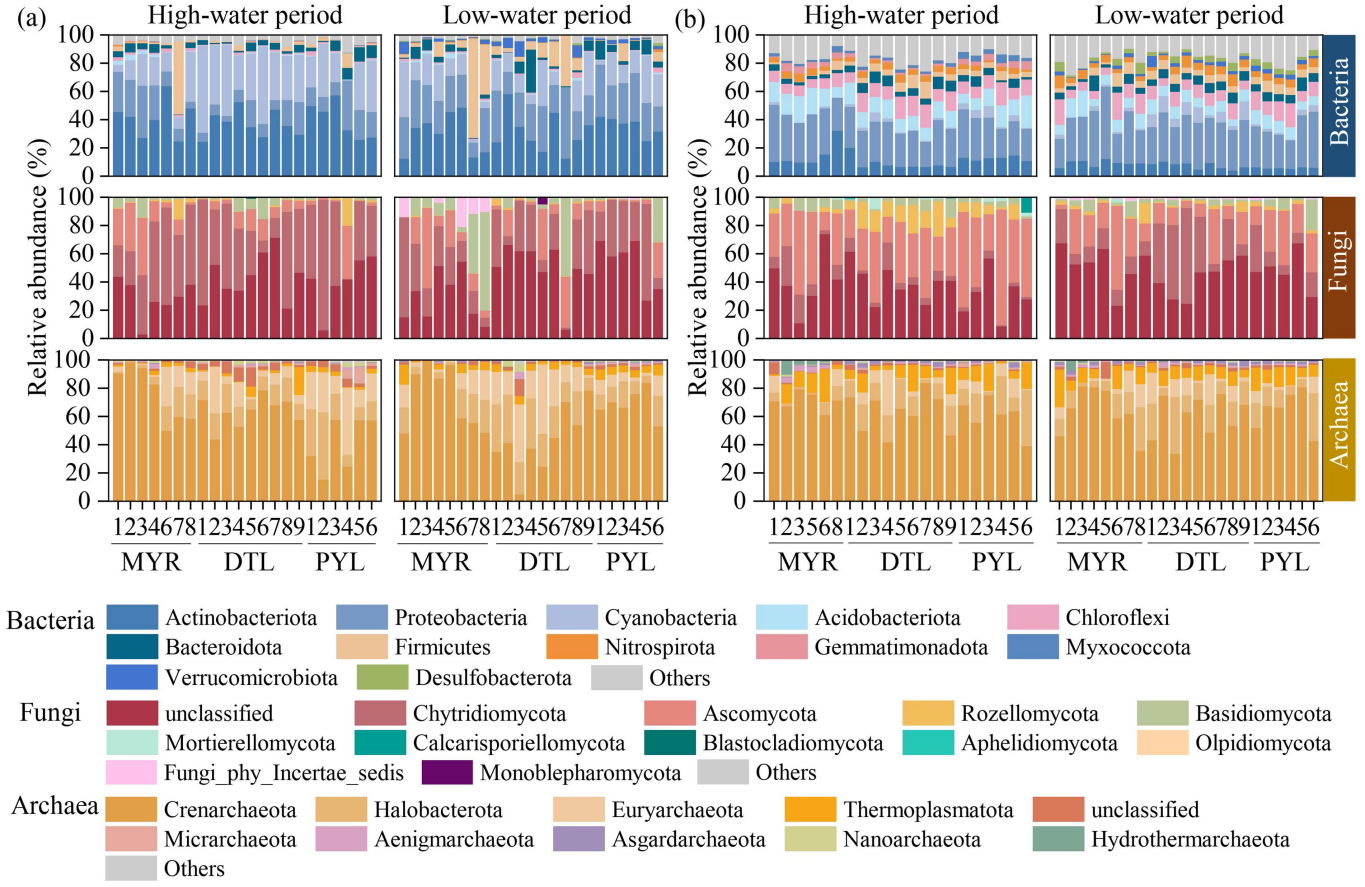
Relative abundance of bacterial, archaeal, and fungal communities at a phyla level in water **(a)** and sediment **(b)** samples.

The relative abundance of bacterial, archaeal and fungal communities at a phyla level is shown in Figure 3. For planktonic bacteria, Actinobacteriota, Proteobacteria, and Cyanobacteria had the highest relative abundance, with an average relative abundance of about 80% cumulatively (Figure 3a). In sedimentary bacteria, Proteobacteria, Actinobacteriota, Acidobacteriota, and Chloroflexi dominated, with a cumulatively average relative abundance exceeding 60% (Figure 3b). In the river–lake system, DTL and PYL exhibited higher relative abundance of Cyanobacteria in both high-water period (40.5% in DTL and 25.2% in PYL) and low-water period (16.7% in DTL and 18.3% in PYL) than MYR (16.9% in high water period and 16.5% in low-water period). Notably, high abundance of planktonic Firmicutes was detected in MYR7 in high-water period and MYR7, MYR8, DTL6, and DTL7 in low-water period (Figure 3a).

For fungal communities, unclassified, Chytridiomycota, and Ascomycota dominated in water samples, with the relative abundances of 41.0%, 36.3%, and 12.1%, respectively (Figure 3a). A higher relative abundance of Basidiomycota was observed in planktonic fungi in MYR7, MYR8, DTL7, and PYL6 in low-water period. Unclassified and Ascomycota were the dominant phyla in sedimentary fungi (Figure 3b). A higher relative abundance of Rozellomycota was found in sedimentary fungi in DTL in high-water period.

For archaeal communities, Crenarchaeota exhibited higher relative abundance in both planktonic and sedimentary archaea across all areas, ranging from 5.00%–99.03% and 33.66%–83.97%, respectively (Figures 3a,b). Specially, the relative abundance of Euryarchaeota in DTL water was increased in low-water period (1.84%–49.14%) than in high-water period (1.54%–42.93%). In contrast, the relative abundance of Euryarchaeota in PYL water was greatly higher in high-water period (12.45%–47.58%) than in low-water period (2.19%–14.47%).

### 3.4 Ecological processes and distance-decay relationships

**Figure 4:**
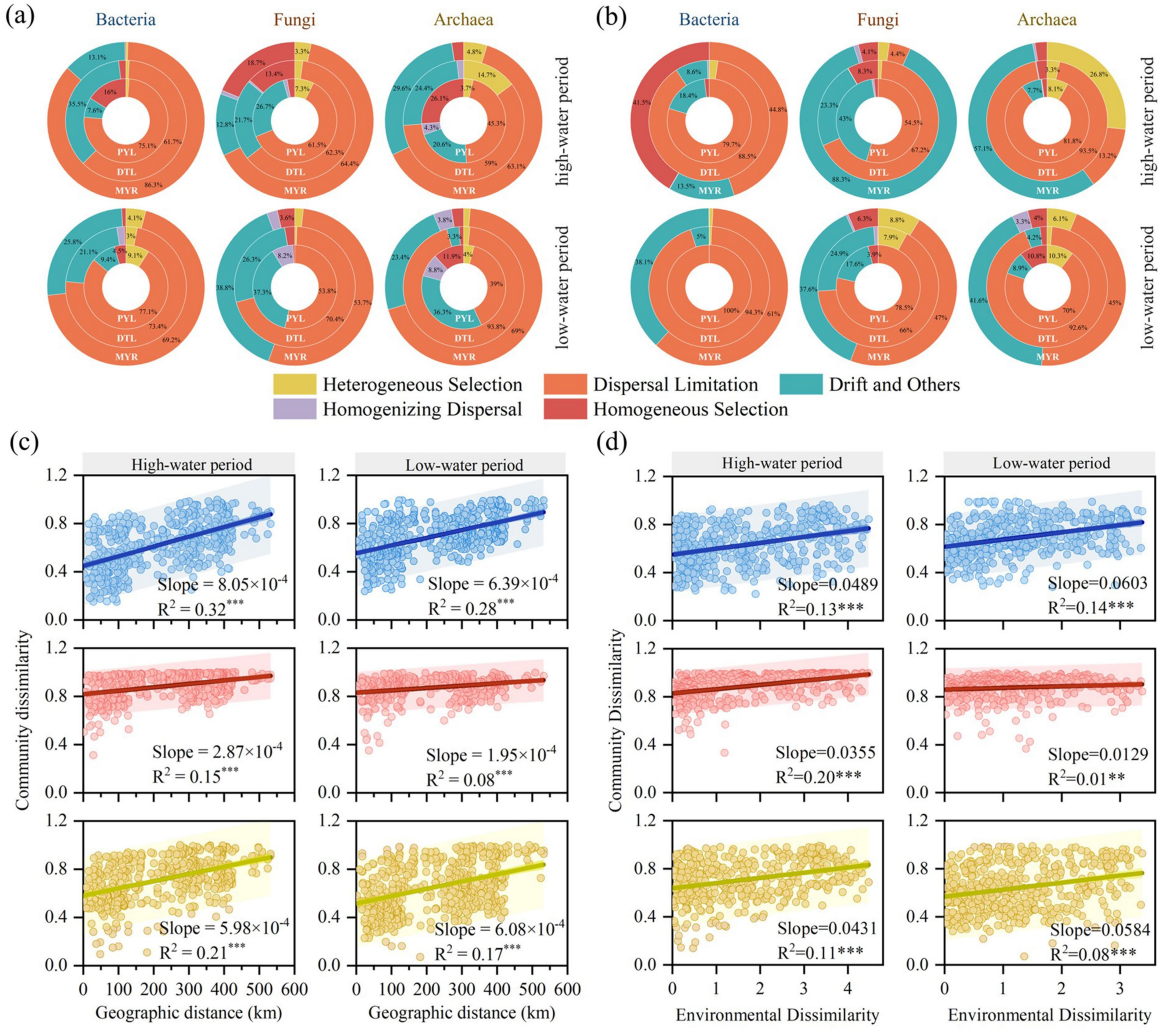
The proportions of individual assembly processes of planktonic **(a)** and sedimentary **(b)** microbial communities in different areas. Distance-decay relationships between microbial communities based on Bray–Curtis similarity and geographic distance **(c)**, environmental variables **(d)**. The environmental dissimilarity was calculated by Euclidean distance. *** means the *p*< 0.001 in linear regression analysis.

The *β*-NRI index was used to evaluate the ecological processes of microbial communities in MYR, DTL, and PYL. Stochastic processes dominated the community assembly of bacteria, fungi and archaea in the river–lake system (Figures 4a,b), with dispersal limitation generally playing a dominant role. In MYR, similar assembly processes were observed for planktonic bacterial, fungal and archaeal communities, with the order of dispersal limitation (53.7%–86.3%) > drift (12.8%–38.8%) (Figure 4a). A similar phenomenon was observed in the assembly mechanisms for bacterial, fungal and archaeal communities in MYR sediment in low period (Figure 4b). However, homogeneous selection (41.5%) become more prominent than drift in shaping the bacterial community, while drift predominantly influenced the fungal and archaeal communities in MYR sediment in high period (Figure 4b). Compared to the MYR, deterministic processes including homogeneous selection and heterogeneous selection had a higher influence in planktonic bacterial and archaea communities in DTL and PYL, but lower influence in planktonic fungal communities (Figure 4a). In DTL and PYL sediment, drift had a greater impact on all communities than in MYR in the two seasons (Figure 4b).

Significant distance-decay relationships were observed in bacterial, fungal and archaeal communities during high-water and low-water period (*p* < 0.0001, Figures 4c,d). With increasing geographic distance and environmental heterogeneity, Bray-Curtis distance (community dissimilarity) significantly increased across all the three microbial communities, indicating that community differentiation was jointly influenced by geographic dispersal limitations and environmental selection (Chen et al., 2020). The effects of geographic distance and environmental variables were higher in bacterial (Slope_Geo-bac_ = 6.39 × 10^−4^–8.05 × 10^−4^, Slope_Env-bac_ = 0.0489–0.0603) and archaeal (Slope_Geo-arc_ = 5.98 × 10^−4^–6.08 × 10^−4^, Slope_Env-arc_ = 0.0431–0.0584) communities compared to fungal community (Slope_Geo-fun_ = 1.95 × 10^−4^–2.87 × 10^−4^, Slope_Env-fun_ = 0.0129–0.0355). Additionally, the relationship between geographic distance and microbial community similarity (*R*^2^_average_ = 0.20) was stronger than that between environmental variables and microbial community similarity (*R*^2^_average_ = 0.11), indicating that geographic distance imposed greater dispersal limitations on microbial communities. At different seasons, the bacterial, fungal and archaeal communities were more significantly influenced by geographic distance and environmental variables in high-water period than in low-water period.

### 3.5 Co-occurrence network

**Figure 5:**
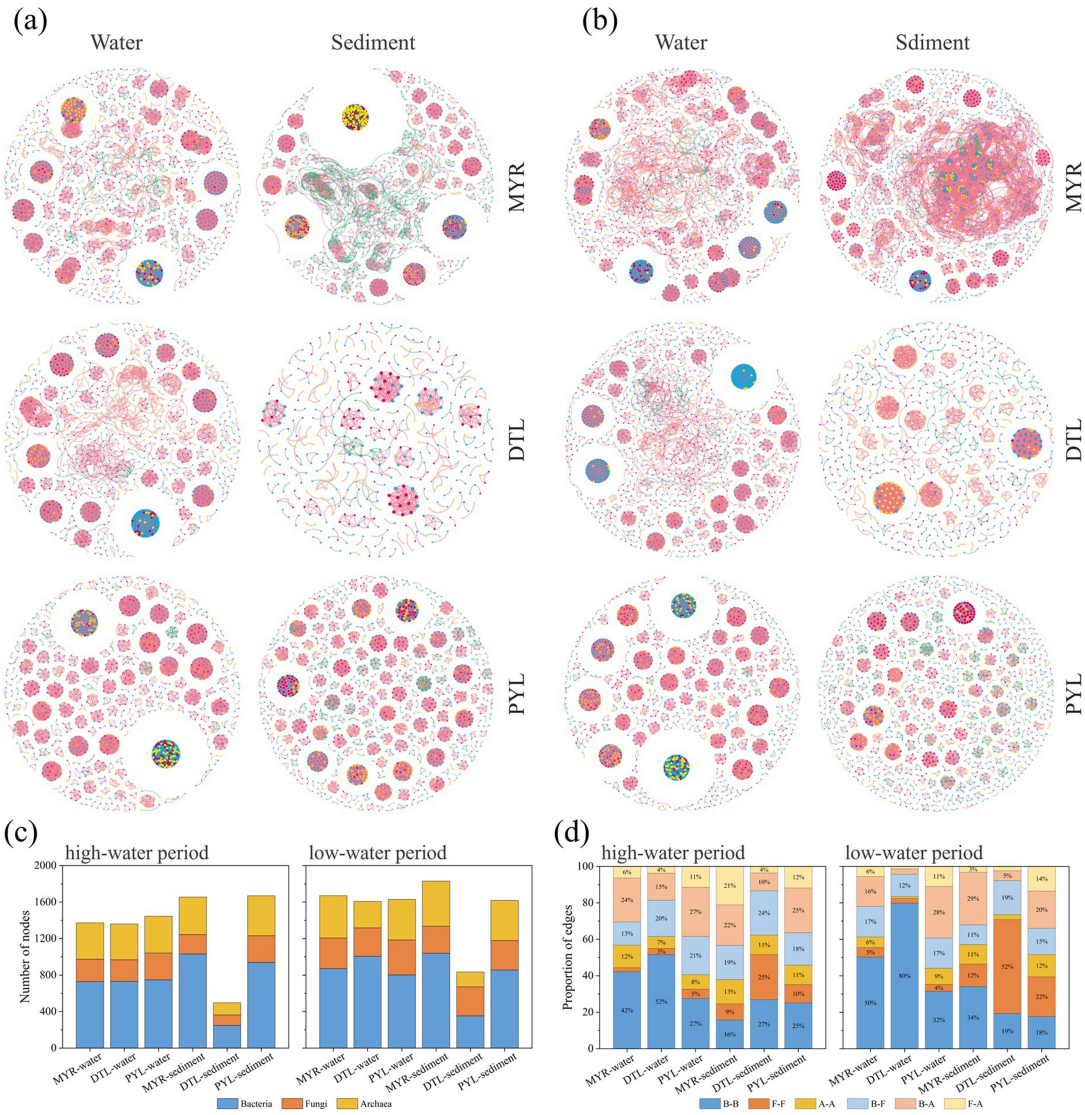
The microbial co-occurrence networks during the high-water period **(a)** and low-water period **(b)** in MYR, DTL, and PYL. The blue, red, and yellow nodes indicate bacterial, fungal and archaeal nodes, while red and green edges represent positive and negative relationships among the OTUs in the network, respectively. The number of bacterial, fungal and archaeal nodes in the networks **(c)**. The proportion of B(bacteria)-B, F(fungi)-F, A(archaea)-A, B-F, B-A and F-A edges in the networks **(d)**.

To explore the symbiotic patterns of bacterial, fungal, and archaeal communities in the river–lake system, we constructed co-occurrence networks for planktonic and sedimentary microbes in high-water and low-water periods (Figures 5a,b). In the co-occurrence networks of different microbial groups, bacterial communities exhibited higher number of nodes than fungi and archaea (Figure 5c). In MYR and DTL, intra-domain cooperation accounted for a higher proportion, while inter-domain cooperation played a dominant role in PYL (Figure 5d). The cooperation between bacteria community accounted for the highest proportion in water, while the proportion of cooperation between fungi community increased significantly in sediments. The network topology parameters showed that the planktonic microbial networks of DTL and PYL had higher network density and lower modularity in both high-water and low-water period, while MYR had higher network density in sedimentary networks (Supplementary Table S4). The Venn diagram (Supplementary Figure S5) showed that there were more overlapped nodes between DTL and MYR than that between PYL and MYR.

**Figure 6:**
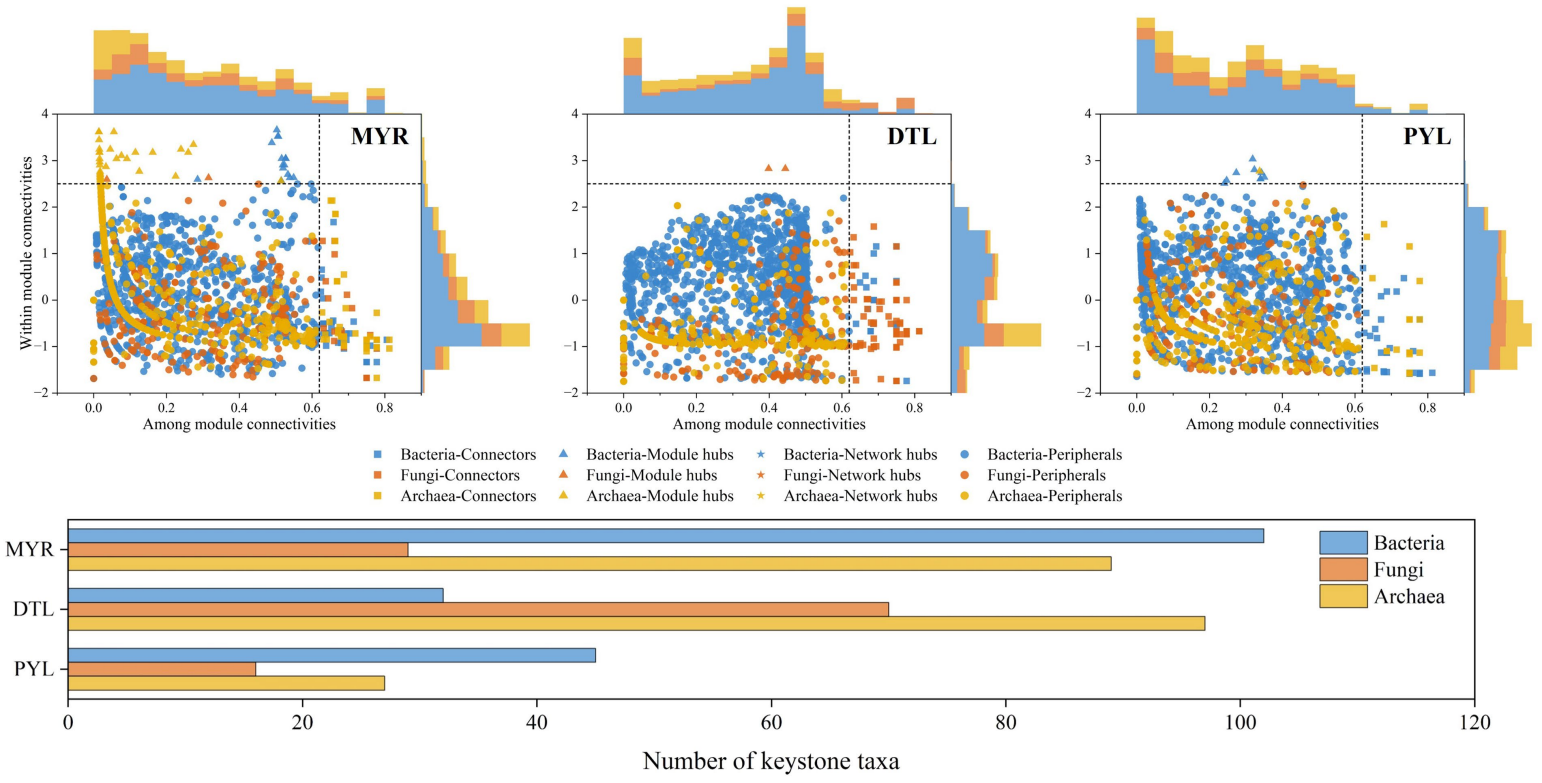
Identification of keystone taxa in bacterial, fungal and archaeal communities during the high-water period based on Zi-Pi.

The Zi-Pi analysis showed that 220, 199, and 88 OTUs were identified as keystone taxa in MYR, DTL, and PYL, respectively, during high-water period (Figure 6), while 273, 205, and 271 OTUs were identified as keystone taxa during low-water period (Supplementary Figure S6). Among the keystone taxa in MYR and PYL, bacteria occupied the main position in the two seasons, followed by archaea in high-water period and fungi in low-water period. While in DTL, archaea occupied the main position in both high-water and low-water period. The betweenness centrality of each keystone token was calculated (Vick-Majors et al., 2014), and the top 20 OTUs in each area were selected and shown in Supplementary Table S5. In MYR, the key bacterial taxa belonged to low-abundance phyla such as *GAL15*, *SAR324_cladeMarine_group* and *MBNT15*. In DTL, the keystone taxa were primarily from the phyla Acidobacteriota and Bacteroidota among bacteria, Basidiomycota and Rozellomycota among fungi and Crenarchaeota among archaea, which were characterized by higher abundance. In PYL, the main keystone taxa in high-water period consisted of 16 bacteria and 4 fungi, while in low-water period, they included eight bacteria, 2 fungi and 10 archaea.

## 4 Discussion

### 4.1 The differentiation of community structure driven by hydrodynamic conditions and environmental factors

Notable differences in microbial community diversity and composition were observed between MYR and its connected lakes. The *α* diversity of bacteria, fungi, and archaea in sediment was significantly higher than that in the water (Supplementary Figure S3), which had the same response of different α diversity indices. Among all bacterial OTUs, only 13.7% were exclusive to planktonic bacteria, whereas this proportion reached 68.3% in sedimentary bacteria. It was consistent with the results of previous studies conducted in the entire Yangtze River Basin (Liu et al., 2018) and the Mississippi River (Staley et al., 2016), emphasizing that sedimentary microbial communities are the primary contributors to microbial diversity in rivers and river-connected lakes. In addition, higher α diversity was observed in DTL sedimentary bacteria, fungi, archaea and in PYL sedimentary bacteria compared to MYR (Figure 2a), because that the longer water retention time in lakes favors the colonization and growth of microbial communities. A previous study reported that the middle Yangtze River had a stable flow velocity of approximately 1.2 m/s, whereas the water flow velocity in river-connected lakes was only 0.2 –0.3 m/s (Lai et al., 2025). Therefore, the high flow velocity of the Yangtze River’s main channel creates a scouring effect on the riverbed, preventing the proliferation and aggregation of microorganisms in localized areas, which is unfavorable for the formation of rich microbial communities (Lai et al., 2025). But the connectivity between the river and river-connected lakes facilitates the migration and dispersal of planktonic microorganisms, contributing to the insignificant differences in planktonic microbial diversity (Figure 2a).

Differences in flow velocity between the river and river-connected lakes also led to variations in microbial community composition. The high flow velocity and low suspended particulate matter in the Yangtze River’s mainstream favored the dominance of rapidly growing bacterial phyla such as Actinobacteriota and Proteobacteria (Liu et al., 2023). Actinobacteriota and Proteobacteria were dominant in the planktonic and sedimentary microbial communities, respectively, which is consistent with previous findings (Staley et al., 2013; Zhang et al., 2020). The members of these two phyla actively participate in various biogeochemical cycles within aquatic ecosystems (Zhang et al., 2013). In contrast, the slower water flow, sediment resuspension and nutrient retention in river-connected lakes promoted the proliferation of Cyanobacteria (Cao et al., 2017) and archaea, particularly Crenarchaeota (Yue et al., 2022) (Figure 3). Notably, Cyanobacteria were abundantly detected in river-connected lakes, especially in DTL during summer (Figure 3b), due to the sufficient sunlight and suitable water temperature in summer (Supplementary Figure S1c). It was reported that suitable temperatures (15.1 °C–35 °C, and reaches peak at 33 °C) could greatly promote the growth of Cyanobacteria (Li et al., 2016). In addition, the main body of DTL exhibits clear spatial partitioning, including West, South, and East DTL with narrow waterway connecting, which leads to extended water retention time in each lake area (Gao et al., 2021). Moreover, the significantly higher concentrations of TOC, COD_Mn_, NH_4_^+^-N, and TP in DTL and PYL (Supplementary Figure S2a) resulting from the intensified human activities increases the risk of eutrophication. As reported, PYL is influenced by industrial wastewater discharge (Teng et al., 2010) and agricultural nonpoint source pollution (Li et al., 2023).

A higher relative abundance of Firmicutes was detected, with over 80% belonging to the classes Clostridia and Bacilli at MYR7 (high-water period) and MYR7, MYR8, DTL6, and DTL7 (low-water period). Firmicutes, which are Gram-positive heterotrophic bacteria involved in nutrient cycling, are a major microbial phylum commonly found in sediment of polluted areas, and some species within this phylum have been proposed as effective indicators of fecal contamination (Yildirim et al., 2018). In our study, the relative abundance of Firmicutes increased steadily along DTL5-DTL6-DTL7 in low-water period (Figure 3a), and significantly decreased after merging into the MYR due to the dilution effect of the upstream water. Additionally, it was found that in river-connected lakes, when tributaries flow into PYL (PYL1 and PYL5, respectively) or when river-connected lakes merge with the mainstream (DTL and PYL flow into the Yangtze River at DTL7 and PYL6, respectively), the microbial community composition changed greatly from adjacent sampling sites (Figure 3). This may be due to differences between the microbial communities of the river (tributary) and lake waters. The original microbial composition could be altered upon mixing (Xiao et al., 2024).

### 4.2 Driving factors and ecological mechanism of microbial community assembly

At all sampling sites, the microbial community assembly processes were predominantly governed by stochastic processes, with dispersal limitation playing a dominant role, followed by drift and homogeneous selection (Figures 4a,b). This suggests that the migration barriers on the spatial scale and the random increase or decrease in microbial population sizes (stochastic birth, death, or migration) is crucial for community assembly. This finding is consistent with previous findings (Chen et al., 2023). For example, the assembly of planktonic bacterial communities in 49 lakes in Paris, France were controlled by stochastic processes (Roguet et al., 2015). In streams and lakes in southern Sweden, stochastic processes explained up to 85% of the microbial community assembly (Östman et al., 2009). The planktonic bacterial communities were significantly less influenced by homogeneous selection compared to fungal and archaeal communities. This can be attributed to the fact that bacteria generally prioritize rapid reproduction and resource acquisition, exhibiting broad environmental adaptability without reliance on specific ecological niches (Malard et al., 2022). In contrast, fungi and archaea tend to have stable survival and competition within specific environments, occupying narrower ecological niches, which makes them more susceptible to dispersal limitation and heterogeneous selection (Tang et al., 2025).

River-connected lakes are extensions of the river, whose water volume and quality are influenced by inflows from tributaries and regulation from the mainstream. Despite having lower water flow velocities compared to the mainstream, river-connected lakes still maintain considerable water movement and short lake water residence time. Consequently, microbial community assembly in these lakes remains primarily governed by stochastic processes. A study in Danjiangkou Reservoir, a typical river-connected lake, revealed that eukaryotic plankton were predominantly influenced by stochastic processes, due to the interconnected habitats characterized by frequent water exchange with the water diversion project (Zhu et al., 2025). However, studies in Bosten Lake (Tang et al., 2020) and Chaohu Lake (Zhang et al., 2020) reported that deterministic processes dominated in isolated lake ecosystems, likely due to the distinct environmental conditions in these lakes. Bosten Lake is a typical inland saline lake located in northwest China, where the high salinity imposes strong selective pressure on microbial communities. Chaohu Lake, although situated in the middle and lower reaches of the Yangtze River, is not directly connected to the river. It is heavily affected by human activities and suffers from severe pollution and eutrophication. Despite its connection to other water systems through numerous tributaries, the slow water renewal rate leads to persistent environmental stress, making deterministic processes more influential in shaping microbial communities (Zhang et al., 2020).

Our study found that the explanatory power of geographic distance on microbial community differences (*R*^2^ = 0.08–0.32, Figure 4c) was significantly higher than that of environmental variables (*R*^2^ = 0.01–0.20, Figure 4d), highlighting the role of dispersal limitation in community assembly. These results showed that the planktonic microbial communities were affected by dispersal limitation in both river and river-connected lakes (Figure 4a). This phenomenon may be attributed to the strong fluidity in both river and river-connected lakes. As water flows downstream, microorganisms are transported via water currents (Liu et al., 2024), and the increasing geographic distance amplifies the difficulty of microbial dispersal. The geographic distance-decay relationships of bacteria and archaea were more significant than that of fungi. This can be mainly attributed to two reasons: (1) the spores produced by fungi can migrate with river for a long distance, reducing the impact of geographic distance on community similarity (Heaton et al., 2020; Zhang S. et al., 2022); (2) the proportion of fungi successfully annotated was significantly lower than that of bacteria and archaea, leading to weak fungal community differences among different sampling sites. Nevertheless, environmental variables still played a role in shaping community composition through localized selection pressures. As the river mainstream flows different landscapes and ecosystems, environmental variables are more susceptible to variations caused by changes in elevation, inflowing tributaries and human activities. These factors lead to more significant environmental disturbances, which contribute to the formation of distinct microbial communities (Doherty et al., 2017).

### 4.3 Co-occurrence network characteristics of bacteria, fungi and archaea

The complexity and connectivity of planktonic microbial co-occurrence networks were significantly higher in river-connected lakes compared to the MYR (Figures 5a,b). In high-water period, PYL exhibited the highest network density and the largest number of edges, suggesting stronger functional redundancy and cooperative interactions (Röttjers and Faust, 2018). This may be attributed to the relatively stable habitat and higher resource availability in river-connected lakes. The robustness analysis (Supplementary Figure S7) exhibited that the bacterial and fungal communities in river-connected lakes has a higher network stability, which supports the findings. In contrast, the lower network density observed in the MYR reflect the characteristics of stochastic community assembly, which is consistent with the ecological instability caused by frequent hydrological disturbances. Intra-domain cooperation dominated in MYR and DTL, while inter-domain cooperation dominated in PYL. Additionally, more overlapped nodes between DTL and MYR than that between PYL and MYR due to the different recharge relationships between the two lakes and MYR. DTL receives part of the water from the main stream of the Yangtze River (Liao et al., 2023), resulting similar networks with MYR. However, the backflow from the Yangtze River to PYL rarely happens in recent years due to the riverbed descending of the Yangtze River (Dai and Liu, 2013), which makes PYL a unidirectional-flowing lake, thus leading to lower similar networks with MYR.

The Zi-Pi analysis showed that there were significant differences in keystone taxa between MYR and river-connected lakes. In MYR, most of the keystone taxa were rare taxa (relative abundance <0.1% in all samples) (Dai et al., 2022), whereas in river-connected lakes, some keystone taxa were abundant taxa (relative abundance ≥0.1% in over 50% samples, and occurred in more than 80% samples) (Dai et al., 2022) (Supplementary Table S5). Although rare species have low abundance within microbial communities (less than 0.1%), they support vulnerable functions due to high functional redundancy (Mace et al., 2013; Zhang et al., 2024). This further supports the predominant role of stochastic processes in microbial community assembly. The betweenness centrality analysis found the keystone taxa (Supplementary Table S5), which played important roles in ecosystem like photosynthesis, nitrification and denitrification. The ecological functions performed by these keystone taxa are of great ecological importance, contributing to the stability of microbial communities.

### 4.4 Limitations and future directions

This study has limitations. First, the eDNA sample collection was conducted only during the high-water and low-water, and multi-seasonal sampling may be needed to comprehensively assess the interannual variations in microbial communities. Second, although we highlighted the influence of human activities on microbial communities, quantification of the contributions of various anthropogenic stressors, such as industrial wastewater discharge and agricultural non-point source pollution is still challenging. However, we avoided high-pollution areas during the sampling campaign, such as wastewater treatment plants and chemical factories, to reduce the spatial heterogeneity caused by point source pollution. This study mainly focused on the spatial distribution characteristics of microorganisms, with an emphasis on comparing bacterial, fungal, and archaeal community differences caused by geographic distance and hydrological features between the river and river-connected lakes. The interannual variation of microbial communities and the influence of anthropogenic pollution on bacterial, fungal, and archaeal communities in the Yangtze River mainstream and river-connected lakes need further studies. Third, our study primarily focused on the taxonomic and assembly-based perspectives of microbial communities. While our findings on community composition, keystone taxa, and network interactions provide strong implicit evidence for potential functional differences, direct verification through metagenomic or metatranscriptomic analyses is warranted in the future to explicitly link the observed structural patterns to ecosystem functions, such as nutrient cycling and organic matter degradation. Such functional insights will be crucial for a predictive understanding of how river–lake ecosystems respond to anthropogenic and natural disturbances.

## 5 Conclusion

This study reveals the assembly and network characteristics of bacterial, fungal and archaeal communities using eDNA technology in a typical hydrologically-connected river–lake ecosystem. Significant spatial heterogeneity in microbial diversity and community composition was found between the MYR and its connected lakes of DTL and PYL, revealing that hydrological connectivity and flow dynamics are critical drivers of microbial community structure in this ecosystem. Stochastic processes, particularly dispersal limitation, dominated the microbial assembly across all sites. The high flow velocity in the MYR limited sedimentary microbial diversity, while the greater water retention in the lakes supported richer communities. DTL, with its strong hydrological exchange with the river, exhibited network traits and intra-domain cooperation similar to MYR. In contrast, PYL, characterized by weaker connectivity and predominantly unidirectional flow, developed distinct inter-domain cooperation and lower species overlap with the MYR.

This study provides broader implications for the conservation and management of large river–lake ecosystems. Our findings underscore the importance of maintaining natural hydrological connectivity to preserve microbial diversity and ecosystem functioning, which are essential for nutrient cycling, pollutant degradation, and overall ecosystem health. The distinct microbial assembly patterns and network structures observed between the river and lakes also suggest that hydrological regulation strategies should consider their potential impacts on microbial communities and associated ecological processes. Furthermore, this study demonstrates the utility of eDNA-based approaches for monitoring microbial responses to environmental changes, offering a valuable tool for assessing ecosystem health under anthropogenic pressures. These insights are not only relevant for the Yangtze River basin but also applicable to other large river–lake systems worldwide, supporting future efforts in ecological restoration, biodiversity conservation, and sustainable water resource management under global change scenarios.

## Data Availability

The original contributions presented in the study are included in the article/Supplementary material, further inquiries can be directed to the corresponding authors.

## References

[ref1] Bai C. Cai J. Zhou L. Jiang X. Hu Y. Dai J.. (2020). Geographic patterns of bacterioplankton among lakes of the middle and lower reaches of the Yangtze River basin, China. Appl. Environ. Microbiol. 86, e02423–e02419. doi: 10.1128/AEM.02423-19, PMID: 31924617 PMC7054100

[ref2] Cao X. Wang J. Liao J. Gao Z. Jiang D. Sun J.. (2017). Bacterioplankton community responses to key environmental variables in plateau freshwater lake ecosystems: a structural equation modeling and change point analysis. Sci. Total Environ. 580, 457–467. doi: 10.1016/j.scitotenv.2016.11.143, PMID: 28040220

[ref3] Caporaso J. G. Kuczynski J. Stombaugh J. Bittinger K. Bushman F. D. Costello E. K.. (2010). QIIME allows analysis of high-throughput community sequencing data. Nat. Methods. 7, 335–336. doi: 10.1038/nmeth.f.303, PMID: 20383131 PMC3156573

[ref4] Chen W. Ma P. Zhang J. Yang Y. Zhou L. Zhao X. (2023). Spatial distribution pattern and effecting factors of aquatic bacteria in Lake Dongting and Lake Poyang. J. Lake Sci. 35, 257–266.

[ref5] Chen J. Wang P. Wang C. Wang X. Miao L. Liu S.. (2020). Fungal community demonstrates stronger dispersal limitation and less network connectivity than bacterial community in sediments along a large river. Environ. Microbiol. 22, 832–849. doi: 10.1111/1462-2920.14795, PMID: 31469494

[ref6] Chen X. Ye Q. Du J. Zhang J. (2019). Bacterial and archaeal assemblages from two size fractions in submarine groundwater near an industrial zone. Water 11:1261. doi: 10.3390/w11061261

[ref7] Chen S. Zhou Y. Chen Y. Gu J. (2018). fastp: an ultra-fast all-in-one FASTQ preprocessor. Bioinformatics 34, i884–i890. doi: 10.1093/bioinformatics/bty560, PMID: 30423086 PMC6129281

[ref8] Dai Z. Liu J. T. (2013). Impacts of large dams on downstream fluvial sedimentation: an example of the three gorges dam (TGD) on the Changjiang (Yangtze River). J. Hydrol. 480, 10–18. doi: 10.1016/j.jhydrol.2012.12.003

[ref9] Dai T. Wen D. Bates C. T. Wu L. Guo X. Liu S.. (2022). Nutrient supply controls the linkage between species abundance and ecological interactions in marine bacterial communities. Nat. Commun. 13:175. doi: 10.1038/s41467-021-27857-6, PMID: 35013303 PMC8748817

[ref10] Doherty M. Yager P. L. Moran M. A. Coles V. J. Fortunato C. S. Krusche A. V.. (2017). Bacterial biogeography across the Amazon River-ocean continuum. Front. Microbiol. 8:882. doi: 10.3389/fmicb.2017.00882, PMID: 28588561 PMC5440517

[ref11] Edgar R. C. (2010). Search and clustering orders of magnitude faster than BLAST. Bioinformatics 26, 2460–2461. doi: 10.1093/bioinformatics/btq461, PMID: 20709691

[ref12] Edgar R. C. (2013). UPARSE: highly accurate OTU sequences from microbial amplicon reads. Nat. Methods. 10, 996–998. doi: 10.1038/nmeth.2604, PMID: 23955772

[ref13] Gao Y. Zhang W. Li Y. Wu H. Yang N. Hui C. (2021). Dams shift microbial community assembly and imprint nitrogen transformation along the Yangtze River. Water Res. 189:116579. doi: 10.1016/j.watres.2020.116579, PMID: 33160238

[ref14] Gill A. S. Purnell K. Palmer M. I. Stein J. McGuire K. L. (2020). Microbial composition and functional diversity differ across urban green infrastructure types. Front. Microbiol. 11:912. doi: 10.3389/fmicb.2020.00912, PMID: 32582043 PMC7291602

[ref15] Gu Y. Li J. Liu Z. Zhang M. Yang Z. Yin H.. (2024). Different adaption strategies of abundant and rare microbial communities in sediment and water of east Dongting Lake. J. Microbiol. 62, 829–843. doi: 10.1007/s12275-024-00171-8, PMID: 39438387

[ref16] Hannula S. E. Morrien E. de Hollander M. van der Putten W. H. van Veen J. A. de Boer W. (2017). Shifts in rhizosphere fungal community during secondary succession following abandonment from agriculture. ISME J. 11, 2294–2304. doi: 10.1038/ismej.2017.90, PMID: 28585935 PMC5607372

[ref17] Heaton L. L. M. Jones N. S. Fricker M. D. (2020). A mechanistic explanation of the transition to simple multicellularity in fungi. Nat. Commun. 11:2594. doi: 10.1038/s41467-020-16072-4, PMID: 32444651 PMC7244713

[ref18] Huang A. Liu X. Peng W. Dong F. Ma B. Li J.. (2022). Spatiotemporal characteristics, influencing factors and evolution laws of water exchange capacity of Poyang Lake. J. Hydrol. 609:127717. doi: 10.1016/j.jhydrol.2022.127717

[ref19] Ji F. Han D. Yan L. Yan S. Zha J. Shen J. (2022). Assessment of benthic invertebrate diversity and river ecological status along an urbanized gradient using environmental DNA metabarcoding and a traditional survey method. Sci. Total Environ. 806:150587. doi: 10.1016/j.scitotenv.2021.150587, PMID: 34582852

[ref20] Kraemer S. A. Barbosa da Costa N. Shapiro B. J. Fradette M. Huot Y. Walsh D. A. (2020). A large-scale assessment of lakes reveals a pervasive signal of land use on bacterial communities. ISME J. 14, 3011–3023. doi: 10.1038/s41396-020-0733-0, PMID: 32770118 PMC7784917

[ref21] Lai X. Liang Q. Jiang J. Huang Q. (2014). Impoundment effects of the three-gorges-dam on flow regimes in two China’s largest freshwater lakes. Water Resour. Manag. 28, 5111–5124. doi: 10.1007/s11269-014-0797-6

[ref22] Lai X. Zou H. Jiang J. Jia J. Liu Y. Wei W. (2025). Hydrological dynamics of the Yangtze river-Dongting lake system after the construction of the three gorges dam. Sci. Rep. 15:50. doi: 10.1038/s41598-024-83751-3, PMID: 39747193 PMC11696283

[ref23] Li F. Peng Y. Fang W. Altermatt F. Xie Y. Yang J.. (2018). Application of environmental DNA metabarcoding for predicting anthropogenic pollution in Rivers. Environ. Sci. Technol. 52, 11708–11719. doi: 10.1021/acs.est.8b03869, PMID: 30211550

[ref24] Li H. Wang S. Che F. Jiahng X. Niu Y. (2023). Mate analysis of heavy metal pollution in sediments of Chaohu Lake, Dongting Lake and Poyang Lake. China Environ. Sci. 43, 831–842. doi: 10.19674/j.cnki.issn1000-6923.20221115.001

[ref25] Li Y. Xie X. Zhu X. Hang X. Li X. Jing Y. (2016). Applying remote sensing techniques in analysis of temperature features causing cyanobacteria bloom in Lake Taihu. J. Lake Sci. 28, 1256–1264. doi: 10.18307/2016.0611

[ref26] Liao W. Tong D. Nie X. Liu Y. Ran F. Liao S.. (2023). Assembly process and source tracking of microbial communities in sediments of Dongting Lake. Soil Ecol. Lett. 5:230173. doi: 10.1007/s42832-023-0173-7

[ref27] Liao H. Yen J. Y. Guan Y. Ke D. Liu C. (2020). Differential responses of stream water and bed sediment microbial communities to watershed degradation. Environ. Int. 134:105198. doi: 10.1016/j.envint.2019.105198, PMID: 31704564

[ref28] Liu Q. Chang F. Xie P. Zhang Y. Duan L. Li H.. (2023). Microbiota assembly patterns and diversity of nine plateau lakes in Yunnan, southwestern China. Chemosphere 314:137700. doi: 10.1016/j.chemosphere.2022.137700, PMID: 36587916

[ref29] Liu Y. Ren Z. Qu X. Zhang M. Yu Y. Zhang Y.. (2020). Microbial community structure and functional properties in permanently and seasonally flooded areas in Poyang Lake. Sci. Rep. 10:4819. doi: 10.1038/s41598-020-61569-z, PMID: 32179796 PMC7076011

[ref30] Liu S. Yu H. Yu Y. Huang J. Zhou Z. Zeng J.. (2022). Ecological stability of microbial communities in Lake Donghu regulated by keystone taxa. Ecol. Indic. 136:108695. doi: 10.1016/j.ecolind.2022.108695

[ref31] Liu X. Zhang L. Wang Y. Hu S. Zhang J. Huang X.. (2024). Microbiome analysis in Asia’s largest watershed reveals inconsistent biogeographic pattern and microbial assembly mechanisms in river and lake systems. iScience 27:110053. doi: 10.1016/j.isci.2024.110053, PMID: 38947525 PMC11214296

[ref32] Liu T. Zhang A. N. Wang J. Liu S. Jiang X. Dang C.. (2018). Integrated biogeography of planktonic and sedimentary bacterial communities in the Yangtze River. Microbiome 6:16. doi: 10.1186/s40168-017-0388-x, PMID: 29351813 PMC5775685

[ref33] Mace G. M. Mouillot D. Bellwood D. R. Baraloto C. Chave J. Galzin R.. (2013). Rare species support vulnerable functions in high-diversity ecosystems. PLoS Biol. 11:e1001569. doi: 10.1371/journal.pbio.1001569 23723735 PMC3665844

[ref34] Magoc T. Salzberg S. L. (2011). FLASH: fast length adjustment of short reads to improve genome assemblies. Bioinformatics 27, 2957–2963. doi: 10.1093/bioinformatics/btr507, PMID: 21903629 PMC3198573

[ref35] Malard L. A. Mod H. K. Guex N. Broennimann O. Yashiro E. Lara E.. (2022). Comparative analysis of diversity and environmental niches of soil bacterial, archaeal, fungal and protist communities reveal niche divergences along environmental gradients in the Alps. Soil Biol. Biochem. 169:108674. doi: 10.1016/j.soilbio.2022.108674

[ref36] Östman Ö. Drakare S. Kritzberg E. S. Langenheder S. Logue J. B. Lindström E. S. (2009). Regional invariance among microbial communities. Ecol. Lett. 13, 118–127. doi: 10.1111/j.1461-0248.2009.01413.x, PMID: 19968693

[ref37] Payne J. T. Jackson C. R. Millar J. J. Ochs C. A. (2020). Timescales of variation in diversity and production of bacterioplankton assemblages in the lower Mississippi River. PLoS One 15:e0230945. doi: 10.1371/journal.pone.0230945, PMID: 32255790 PMC7138331

[ref38] Roguet A. Laigle G. S. Therial C. Bressy A. Soulignac F. Catherine A.. (2015). Neutral community model explains the bacterial community assembly in freshwater lakes. FEMS Microbiol. Ecol. 91:fiv125. doi: 10.1093/femsec/fiv125, PMID: 26472576

[ref39] Röttjers L. Faust K. (2018). From hairballs to hypotheses–biological insights from microbial networks. FEMS Microbiol. Rev. 42, 761–780. doi: 10.1093/femsre/fuy030, PMID: 30085090 PMC6199531

[ref40] Staley C. Gould T. J. Wang P. Phillips J. Cotner J. B. Sadowsky M. J. (2014). Bacterial community structure is indicative of chemical inputs in the upper Mississippi River. Front. Microbiol. 5:524. doi: 10.3389/fmicb.2014.00524, PMID: 25339945 PMC4189419

[ref41] Staley C. Gould T. J. Wang P. Phillips J. Cotner J. B. Sadowsky M. J. (2016). Sediments and soils act as reservoirs for taxonomic and functional bacterial diversity in the upper Mississippi River. Microb. Ecol. 71, 814–824. doi: 10.1007/s00248-016-0729-5, PMID: 26879939

[ref42] Staley C. Unno T. Gould T. J. Jarvis B. Phillips J. Cotner J. B.. (2013). Application of Illumina next-generation sequencing to characterize the bacterial community of the upper Mississippi River. J. Appl. Microbiol. 115, 1147–1158. doi: 10.1111/jam.12323, PMID: 23924231

[ref43] Tang M. Chen Q. Xiao X. Lyu Y. Sun W. (2025). Differential impacts of water diversion and environmental factors on bacterial, archaeal, and fungal communities in the eastern route of the south-to-north water diversion project. Environ. Int. 195:109280. doi: 10.1016/j.envint.2025.109280, PMID: 39824026

[ref44] Tang X. Xie G. Shao K. Hu Y. Cai J. Bai C.. (2020). Contrast diversity patterns and processes of microbial community assembly in a river-lake continuum across a catchment scale in northwestern China. Environ. Microb. 15:10. doi: 10.1186/s40793-020-00356-9, PMID: 33902721 PMC8066441

[ref45] Teng Y. Ni S. Wang J. Zuo R. Yang J. (2010). A geochemical survey of trace elements in agricultural and non-agricultural topsoil in Dexing area, China. J. Geochem. Explor. 104, 118–127. doi: 10.1016/j.gexplo.2010.01.006

[ref46] Vannote R. L. Minshall G. W. Cummins K. W. Sedell J. R. Gushing C. E. (1980). The river continuum concept. Can. J. Fish. Aquat. Sci. 37, 130–137. doi: 10.1139/f80-017

[ref47] Vick-Majors T. J. Priscu J. C. Amaral-Zettler L. A. (2014). Modular community structure suggests metabolic plasticity during the transition to polar night in ice-covered Antarctic lakes. ISME J. 8, 778–789. doi: 10.1038/ismej.2013.190, PMID: 24152712 PMC3960534

[ref48] von Hoyningen-Huene A. J. E. Schneider D. Fussmann D. Reimer A. Arp G. Daniel R. (2019). Bacterial succession along a sediment porewater gradient at Lake Neusiedl in Austria. Sci. Data 6:163. doi: 10.1038/s41597-019-0172-9, PMID: 31471542 PMC6717209

[ref49] Wang Y. Molinos J. G. Shi L. Zhang M. Wu Z. Zhang H.. (2019). Drivers and changes of the Poyang Lake wetland ecosystem. Wetlands 39, 35–44. doi: 10.1007/s13157-019-01180-9

[ref50] Wu B. Tian J. Bai C. Xiang M. Sun J. Liu X. (2013). The biogeography of fungal communities in wetland sediments along the Changjiang River and other sites in China. ISME J. 7, 1299–1309. doi: 10.1038/ismej.2013.29, PMID: 23446835 PMC3695295

[ref51] Xiao P. Wu Y. Zuo J. Grossart H.-P. Sun R. Li G.. (2024). Differential microbiome features in lake–river systems of Taihu basin in response to water flow disturbance. Front. Microbiol. 15:1479158. doi: 10.3389/fmicb.2024.1479158, PMID: 39411429 PMC11475019

[ref52] Xu X. Yuan Y. Wang Z. Zheng T. Cai H. Yi M.. (2023). Environmental DNA metabarcoding reveals the impacts of anthropogenic pollution on multitrophic aquatic communities across an urban river of western China. Environ. Res. 216:114512. doi: 10.1016/j.envres.2022.114512, PMID: 36208790

[ref53] Yang Y. Zhang M. Liu W. Wang J. Li X. (2019). Relationship between waterway depth and low-flow water levels in reaches below the three gorges dam. J. Waterw. Port Coast. Ocean Eng. 145:04018032. doi: 10.1061/(asce)ww.1943-5460.0000482

[ref54] Ye J. Joseph S. D. Ji M. Nielsen S. Mitchell D. R. G. Donne S.. (2017). Chemolithotrophic processes in the bacterial communities on the surface of mineral-enriched biochars. ISME J. 11, 1087–1101. doi: 10.1038/ismej.2016.187, PMID: 28169988 PMC5437921

[ref55] Ye X. Li X. Zhang Q. (2012). Temporal variation of backflow frequency from the Yangtze River to Poyang Lake and its influencing factors. J. Southwest Univ. 34, 69–75. doi: 10.13718/j.cnki.xdzk.2012.11.007

[ref56] Yildirim A. Muñoz-Vargas L. Opiyo S. O. Digianantonio R. Williams M. L. Wijeratne A.. (2018). Fecal microbiome of periparturient dairy cattle and associations with the onset of *Salmonella* shedding. PLoS One 13:e0196171. doi: 10.1371/journal.pone.0196171 29750790 PMC5947886

[ref57] Yue Y. Wang F. Pan J. Chen X.-P. Tang Y. Yang Z.. (2022). Spatiotemporal dynamics, community assembly and functional potential of sedimentary archaea in reservoirs: coaction of stochasticity and nutrient load. FEMS Microbiol. Ecol. 98:fiac109. doi: 10.1093/femsec/fiac109, PMID: 36111740

[ref58] Zhang M. Bai L. Yao Z. Li W. Yang W. (2025). Seasonal lake ice cover drives the restructuring of bacteria-archaea and bacteria-fungi interdomain ecological networks across diverse habitats. Environ. Res. 269:120907. doi: 10.1016/j.envres.2025.120907, PMID: 39848515

[ref59] Zhang Y. Huo Y. Zhang Z. Zhu S. Fan W. Wang X.. (2022). Deciphering the influence of multiple anthropogenic inputs on taxonomic and functional profiles of the microbial communities in Yitong River, Northeast China. Environ. Sci. Pollut. Res. 29, 39973–39984. doi: 10.1007/s11356-021-18386-2, PMID: 35112248

[ref60] Zhang S. Li K. Hu J. Wang F. Chen D. Zhang Z.. (2022). Distinct assembly mechanisms of microbial sub-communities with different rarity along the Nu River. J. Soils Sediment. 22, 1530–1545. doi: 10.1007/s11368-022-03149-4

[ref61] Zhang G. Liu S. Du W. Li Y. Wu Z. Liu T.. (2024). Spatiotemporal distributions, co-occurrence networks, and assembly mechanisms of the bacterial community in sediments of the Yangtze River: comprehensive insights into abundant and rare taxa. Front. Microbiol. 15:1444206. doi: 10.3389/fmicb.2024.1444206, PMID: 39723140 PMC11668926

[ref62] Zhang T. Xu S. Yan R. Wang R. Gao Y. Kong M.. (2022). Similar geographic patterns but distinct assembly processes of abundant and rare bacterioplankton communities in river networks of the Taihu Basin. Water Res. 211:118057. doi: 10.1016/j.watres.2022.118057, PMID: 35066261

[ref63] Zhang J. Zhang X. Liu Y. Xie S. Liu Y. (2013). Bacterioplankton communities in a high-altitude freshwater wetland. Ann. Microbiol. 64, 1405–1411. doi: 10.1007/s13213-013-0785-8

[ref64] Zhang L. Zhao F. Li X. Lu W. (2020). Contribution of influent rivers affected by different types of pollution to the changes of benthic microbial community structure in a large lake. Ecotoxicol. Environ. Saf. 198:110657. doi: 10.1016/j.ecoenv.2020.110657, PMID: 32344267

[ref65] Zhou J. Ning D. (2017). Stochastic community assembly: does it matter in microbial ecology? Microbiol. Mol. Biol. Rev. 81, e00002–e00017. doi: 10.1128/mmbr.00002-17, PMID: 29021219 PMC5706748

[ref66] Zhu L. Feng L. Zhang D. Shi F. Zou X. Yang Q.. (2025). Eukaryotic plankton community and assembly processes in a large-scale water diversion project in China. Sci. Rep. 15:4365. doi: 10.1038/s41598-025-87983-9, PMID: 39910192 PMC11799226

