# Supplementary material for: The assembly processes and network characteristics of bacterial, fungal and archaeal communities in the middle Yangtze River and river-connected lakes
Source: Front Microbiol. 2025 Oct 24;16:1701799. doi: 10.3389/fmicb.2025.1701799 (PMC12592041; doi:10.3389/fmicb.2025.1701799)
Supplement: Supplementary file 2 [file Data_Sheet_1.PDF]

Supplementary Information for

The assembly processes and network characteristics of bacterial, fungal and archaeal communities in the middle Yangtze River and river-connected lakes

Fenglin Wang[1,2], Si Li[3*], Pinjian Li[1,2], Chuanzhe Feng[1,2], Zhijie Zhao[1,2], Yulong Yang[1,2], Fulei Han[1,2], An Xue[1,2], Zhenshan Li[1,2], Peng Han[1,2*]

[1]College of Environmental Sciences and Engineering, Key Laboratory of Water and Sediment Sciences, Ministry of Education, Peking University, Beijing, 100871, China

[2]State Environmental Protection Key Laboratory of All Material Fluxes in River Ecosystems, Beijing, 100871, China

[3]Beijing Key Laboratory of Farmland Soil Pollution Prevention and Remediation, College of Resources and Environmental Sciences, China Agricultural University, Beijing 100193, China

E-mail addresses: sili@cau.edu.cn (S. Li); 0006173258@pku.edu.cn (P. Han)

Text S1. Standards for quality filtering and merging.

(i) The reads were truncated at any site receiving an average quality score of <20 over a 50 bp sliding window, and the truncated reads shorter than 50 bp were discarded, reads containing ambiguous characters were also discarded.

(ii) Only overlapping sequences longer than 10 bp were assembled according to their overlapped sequence. The maximum mismatch ratio of overlap region is 0.2. Reads that could not be assembled were discarded.

(iii) Samples were distinguished according to the barcode and primers, and the sequence direction was adjusted, exact barcode matching, 2 nucleotide mismatch in primer matching.

Text S2. Quantification and statistical analysis.

Based on the OTUs information, rarefaction curves and α diversity indices including observed OTUs, Ace, Chao, Coverage, Shannon, Simpson, Sobs index were calculated with Mothur v1.30.2 (Schloss et al., 2009). The Mann-Whitney U test was used to compare significant differences in environmental variables, number of OTUs, and α diversity indices among different groups. The similarity among the microbial communities in different samples was determined by non-metric multidimensional scaling (NMDS) based on Bray-Curtis dissimilarity. The significance of community compositional differences between different taxonomic groups was further tested by analysis of similarity (ANOSIM). The similarity matrix, NMDS and ANOSIM were conducted in R v4.3.1 (https://www.r-project.org/) with the vegan v2.6-8 (10.32614/CRAN.package.vegan) package. Unless otherwise noted, significant difference was determined at $p < 0.05$, and OTUs with abundance lower than 0.01% were excluded for all statistics analysis. Distance-decay curves were plotted for the microbial community similarity versus geographic distance or environmental heterogeneity. The environmental heterogeneity in water and sediment samples among sampling sites was calculated based on the Euclidean distance in R, while the distance-decay rate was calculated based on Origin 2024.

To investigate the community assembly mechanisms of different biological groups, phylogenetic bin-based null model analysis (iCAMP) was applied with the R package (Ning et al., 2020), quantifying the relative importance of five assembly processes (homogeneous selection, heterogeneous selection, dispersal limitation, homogenizing dispersal and drift). β-nearest taxon index (β-NTI) was calculated to distinguish between deterministic (selection) and stochastic processes, where β-NTI < -2 and β-NTI > 2 was represent homogenous and heterogeneous selection, respectively. We calculated Raup-Crick index (RC) to enable the differentiation of specific stochastic processes, where RC > 0.95, RC < -0.95 and -0.95 < RC < 0.95 was represent dispersal limitation, homogenizing dispersal and drift, respectively (Li et al., 2019; Hu et al., 2020; Wang et al., 2020). The β-NTI and RC were calculated in R with the NST and iCAMP package (Zheng et al., 2022).

The co-occurrence networks are used to study species interactions and clarify the importance of key species (Röttjers and Faust, 2018). To determine the cooperation and competition among microorganisms in different seasons and identify key species in different areas, totally 12 microbial co-occurrence networks were constructed. The steps are as follows: 1) OTUs with relative abundance > 0.01% of total sequences were selected, and pairwise Spearman correlation analysis was performed to calculate abundance variation correlations; 2) Construct the correlation matrix by retaining values with correlation coefficients $r \geq 0.8$ and statistical significance $p < 0.01$; 3) calculate topological parameters of the networks; 4) Identify the top 20 OTUs with betweenness centrality values as key species in each network; 5) Construct the co-occurrence networks. Data analysis was conducted using R and Hmisc v.5.2.1 package (Csárdi and Nepusz, 2006), while network rendering and visualization were implemented with Gephi. The within modular degree (Zi) and among modular degree (Pi) of nodes in the network were determined simultaneously using the module determined with the greedy module optimal algorithm. The average network efficiency (Eff), natural connectivity (Eigen) and critical removal fraction of vertices (Pcr) were considered to assess the network robustness through random node or edge removal.

The average efficiency of the network is defined:

$$Eff = \frac{1}{N(N-1)} \sum_{i \neq j \in G} \frac{1}{d(i,j)} \qquad \text{(S1)}$$

where N is the total number of nodes and $d(i,j)$ is the shortest path between node i and node j. When the weight is found in the edge attributes, $d(i,j)$ denotes the weighted shortest path between node i and node j (Liu et al., 2017; Bellingeri et al., 2020).

The natural connectivity can be regarded as an average eigenvalue that changes strictly monotonically with the addition or deletion of edges. It is defined:

$$\bar{\lambda} = \ln\left(\frac{1}{N} \sum_{i=1}^{N} e^{\lambda i}\right) \qquad \text{(S2)}$$

where λi is the ith eigenvalue of the graph adjacency matrix. The larger the value of $\bar{\lambda}$ is, the more robust the network is (Liu et al., 2017).

Pcr is a robustness measure based on random graph theory. The critical fraction against random attacks is labeled as P_c^r. It is defined:

$$P_c^r = 1 - \frac{1}{\frac{\langle k^2 \rangle}{\langle k \rangle} - 1} \tag{S3}$$

where $\langle k \rangle$ is the average nodal degree of the original network, and $\langle k^2 \rangle$ is the average of square of nodal degree (Paul et al., 2005; Liu et al., 2017).

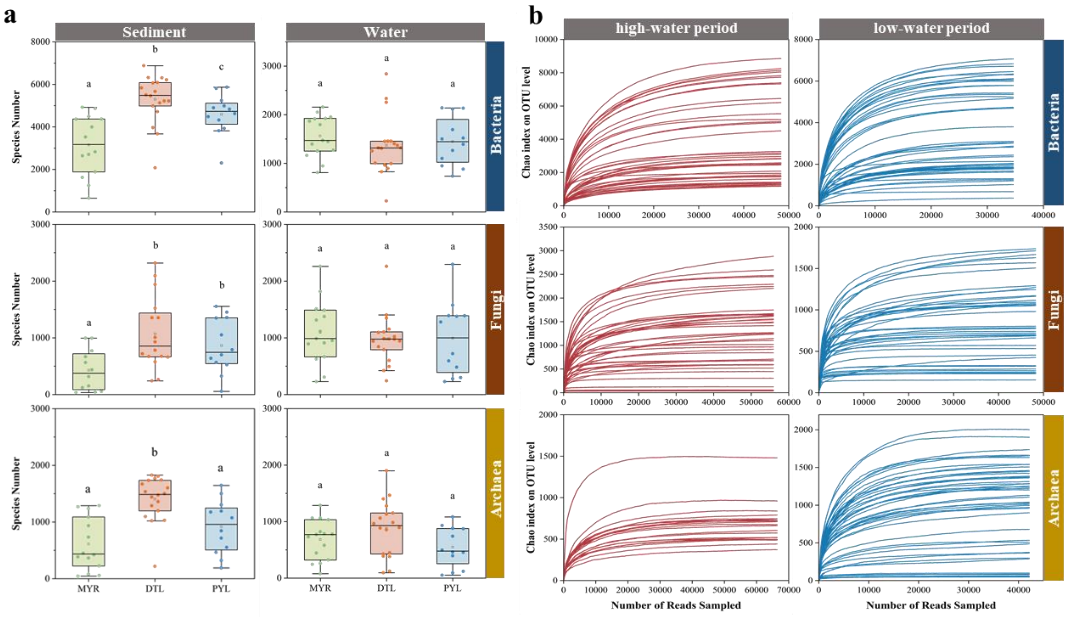

Figure S1. DNA sequencing results. OTU numbers of bacterial, archaeal, and fungal communities in sediment and water samples (a). Rarefaction curves of the 16S/18S rDNA gene reads based on OTUs at 97% sequence similarity (b).

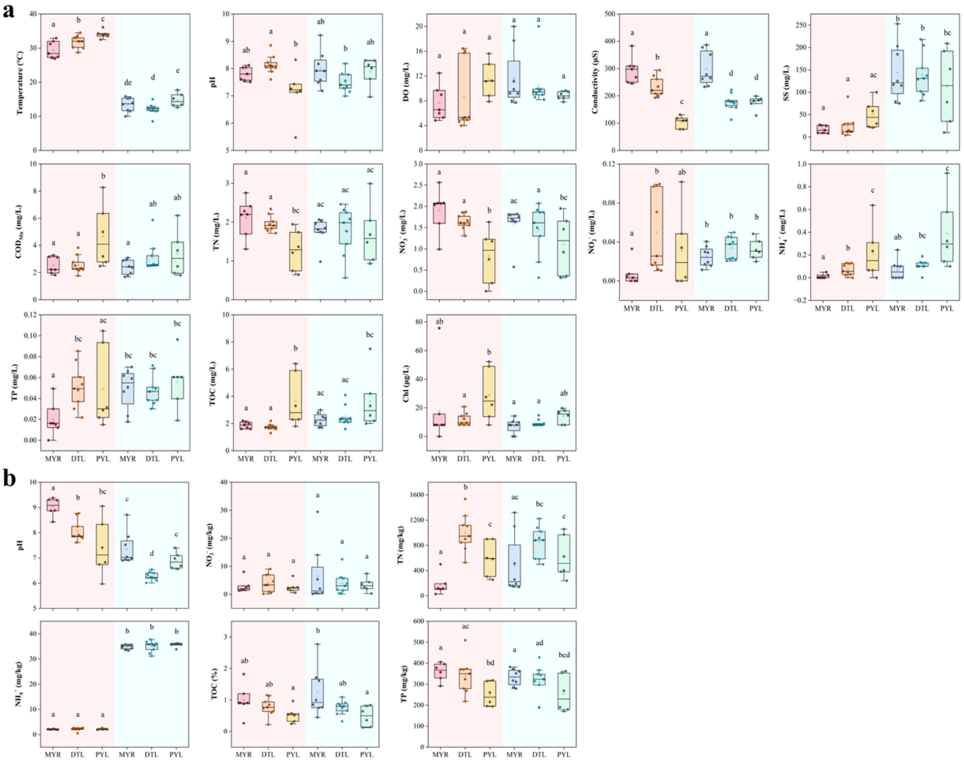

Figure S2. Comparisons of environmental variables of water (a) and sediment (b) in MYR, DTL and PYL. The red zone indicates high-water period, while the blue zone represents low-water period.

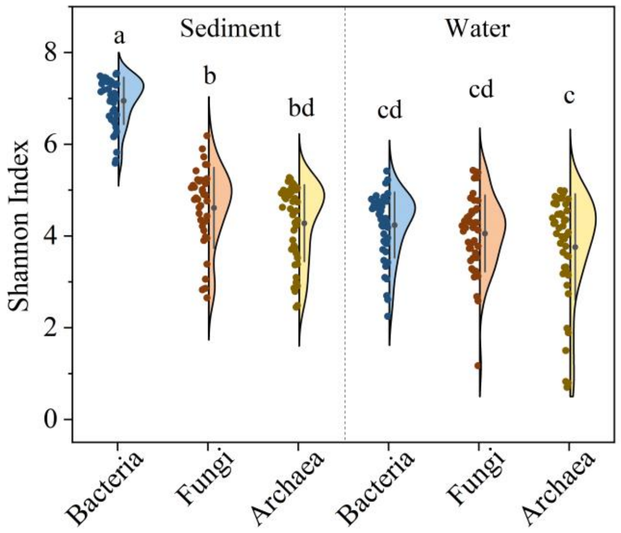

Figure S3. Violin plots of Shannon index of different microbial groups.

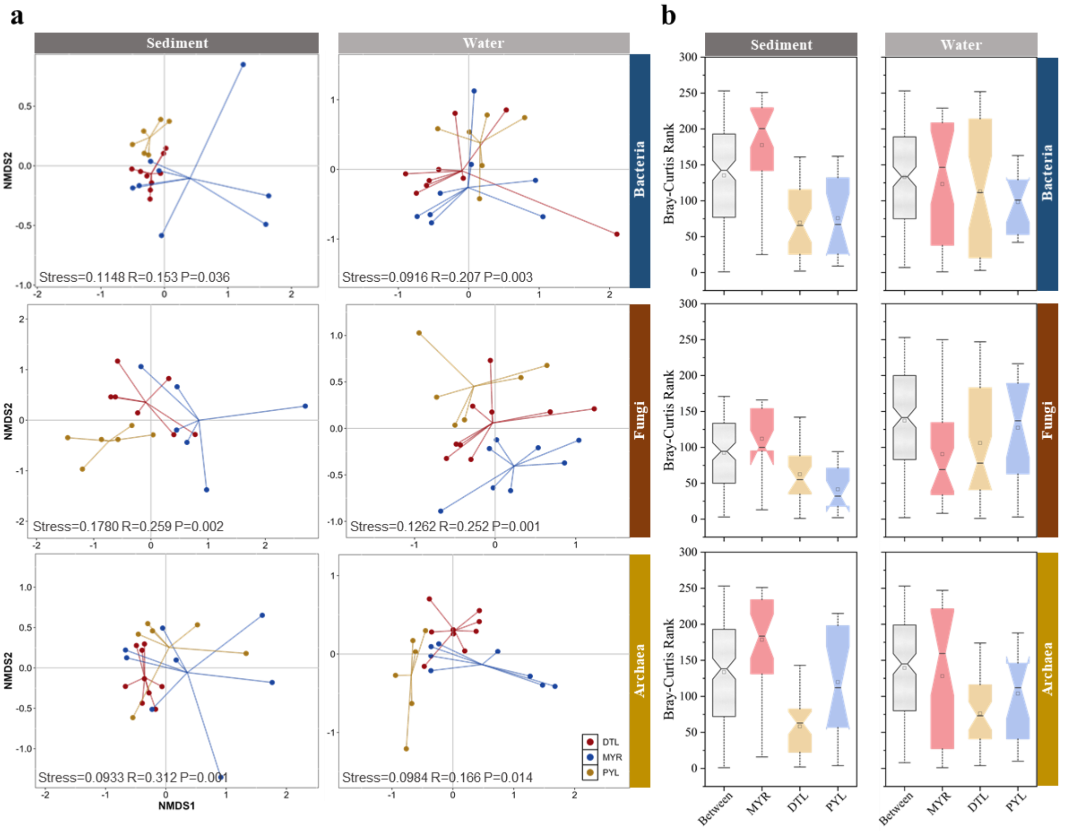

Figure S4. Nonmetric multidimensional scaling (NMDS) analysis based on Bray-Curtis dissimilarity of bacteria, fungi and archaea in low-water period (a). ANOSIM analysis showed compositional differences among MYR, DTL and PYL in low-water period (b).

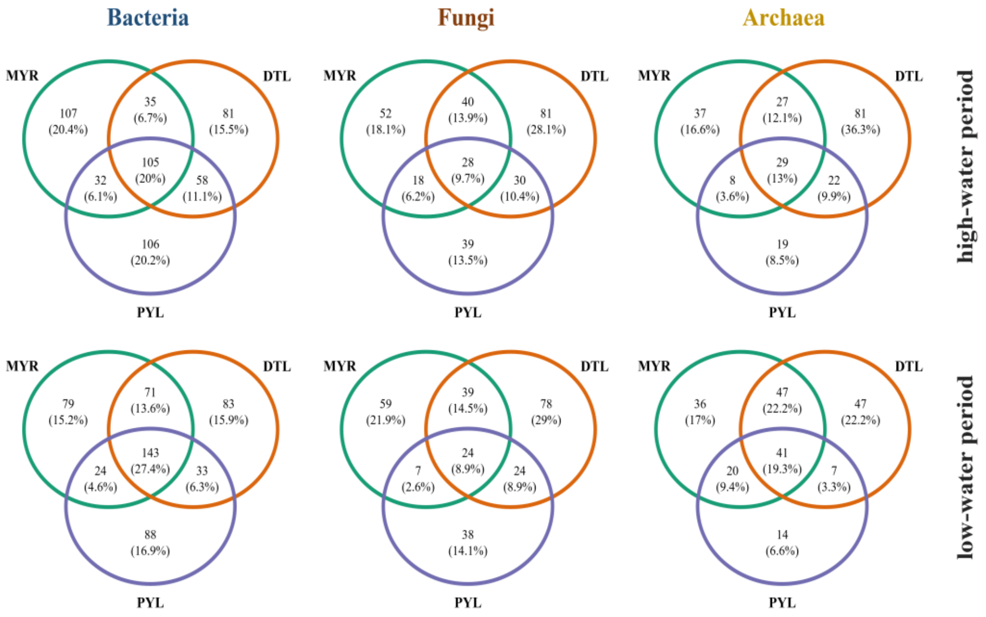

Figure S5. The Venn diagram of node overlap in different regions.

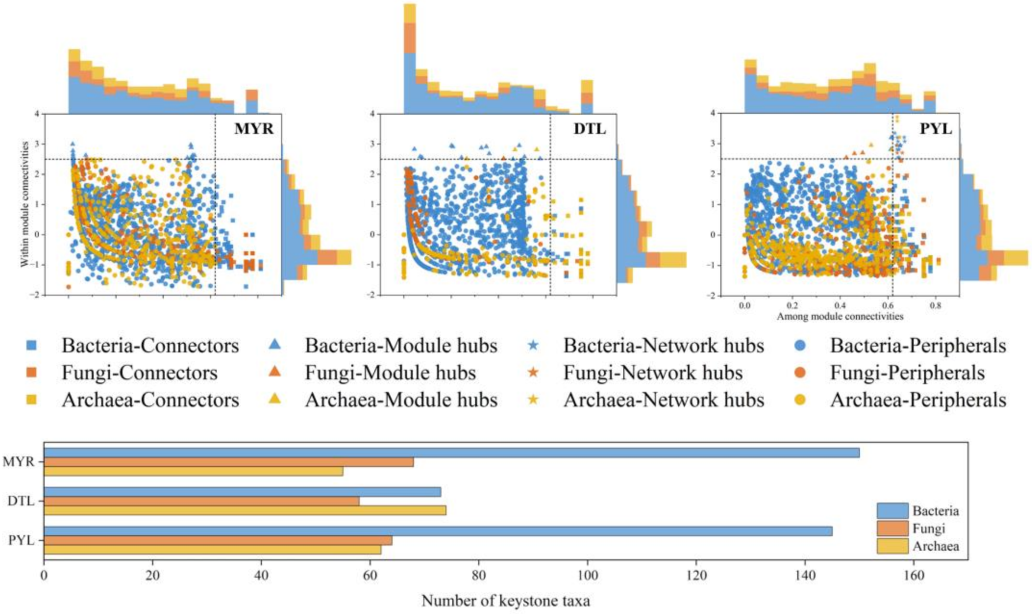

Figure S6. Identification of keystone taxa in bacterial, fungal and archaeal communities during the low-water period based on Zi-Pi.

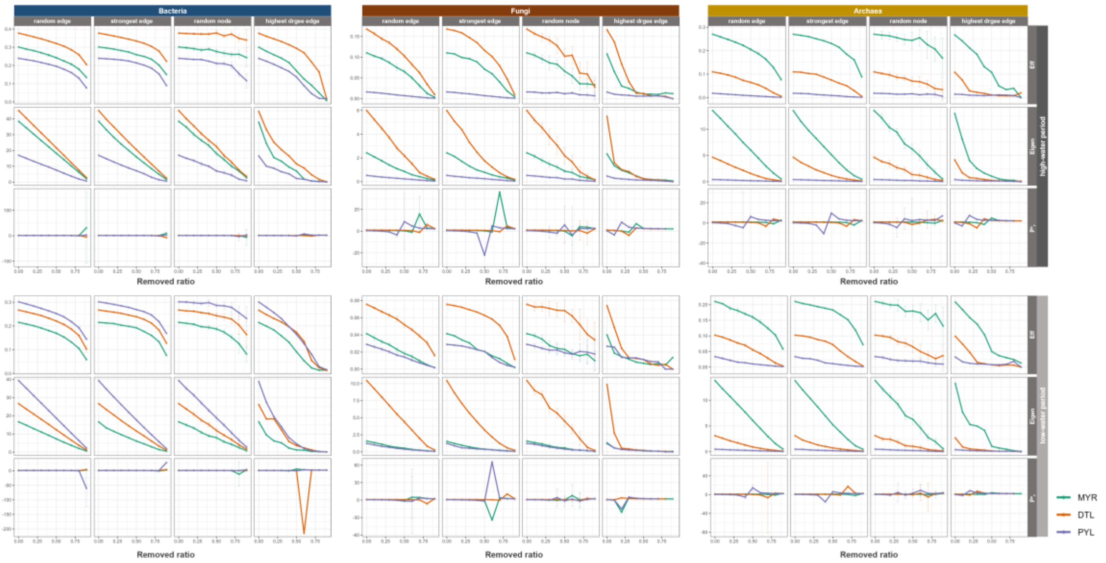

Figure S7. The robustness analysis exhibited the relationships between network efficiency, natural connectivity, critical removal fraction of vertices and the proportion of removed edges or nodes for bacterial, fungal and archaeal networks. The *random edges* indicates that edges are removed randomly, *strongest edge* indicates that edges are removed in decreasing order of weight. The *random node* indicates that nodes are removed randomly, *highest degree node* indicates that nodes are removed in decreasing order of degree.

References

Bellingeri, M., Bevacqua, D., Scotognella, F., Alfieri, R., and Cassi, D. (2020). A comparative analysis of link removal strategies in real complex weighted networks. *Scientific Reports* 10(1). doi: 10.1038/s41598-020-60298-7.

Csárdi, G., and Nepusz, T. (Year). "The igraph software package for complex network research").

Hu, H., He, J., Yan, H., Hou, D., Zhang, D., Liu, L., et al. (2020). Seasonality in Spatial Turnover of Bacterioplankton Along an Ecological Gradient in the East China Sea: Biogeographic Patterns, Processes and Drivers. *Microorganisms* 8(10). doi: 10.3390/microorganisms8101484.

Li, Y., Gao, Y., Zhang, W., Wang, C., Wang, P., Niu, L., et al. (2019). Homogeneous selection dominates the microbial community assembly in the sediment of the Three Gorges Reservoir. *Science of The Total Environment* 690**,** 50-60. doi: 10.1016/j.scitotenv.2019.07.014.

Liu, J., Zhou, M., Wang, S., and Liu, P. (2017). A comparative study of network robustness measures. *Frontiers of Computer Science* 11(4)**,** 568-584. doi: 10.1007/s11704-016-6108-z.

Ning, D., Yuan, M., Wu, L., Zhang, Y., Guo, X., Zhou, X., et al. (2020). A quantitative framework reveals ecological drivers of grassland microbial community assembly in response to warming. *Nature Communications* 11(1). doi: 10.1038/s41467-020-18560-z.

Paul, G., Sreenivasan, S., and Stanley, H.E. (2005). Resilience of complex networks to random breakdown. *Physical Review E* 72(5). doi: 10.1103/PhysRevE.72.056130.

Röttjers, L., and Faust, K. (2018). From hairballs to hypotheses–biological insights from microbial networks. *FEMS Microbiology Reviews* 42(6)**,** 761-780. doi: 10.1093/femsre/fuy030.

Schloss, P.D., Westcott, S.L., Ryabin, T., Hall, J.R., Hartmann, M., Hollister, E.B., et al. (2009). Introducing mothur: Open-Source, Platform-Independent, Community-Supported Software for Describing and Comparing Microbial Communities. *Applied and Environmental Microbiology* 75(23)**,** 7537-7541. doi: 10.1128/aem.01541-09.

Wang, K., Yan, H., Peng, X., Hu, H., Zhang, H., Hou, D., et al. (2020). Community assembly of bacteria and archaea in coastal waters governed by contrasting mechanisms: A seasonal perspective. *Molecular Ecology* 29(19)**,** 3762-3776. doi: 10.1111/mec.15600.

Zheng, L., Wang, X., Ren, M., Yuan, D., Tan, Q., Xing, Y., et al. (2022). Comparing with oxygen, nitrate simplifies microbial community assembly and improves function as an electron acceptor in wastewater treatment. *Environmental Pollution* 314. doi: 10.1016/j.envpol.2022.120243.
